# [Corrigendum] EBM84 attenuates airway inflammation and mucus hypersecretion in an ovalbumin-induced murine model of asthma

**DOI:** 10.3892/ijmm.2026.5963

**Published:** 2026-08-20

**Authors:** In Sik Shin, Mee Young Lee, Woo Young Jeon, Na Ra Shin, Chang Seob Seo, Hyekyung Ha

Int J Mol Med 31: 982-988, 2013; DOI: 10.3892/ijmm.2013.1273

Following the publication of the above article, an interested reader drew the authors' attention to the fact that, in Fig. 4B on p. 987, the 'Mon' and 'EBM84-M' data panels showed apparently the same data, albeit the images were rotated through 180° relative to each other, suggesting that this figure part had been assembled incorrectly.

The authors were able to consult their original data, and acknowledged that errors had been made in compiling Fig. 4B; specifically, the panel labeled as 'Mon' was inadvertently published using the image corresponding to the 'EBM84-H' group; secondly, the panel labeled as 'EBM84-M' incorrectly duplicated the published 'Mon' panel (which, as noted above, was the 'EBM84-H' image); and finally, the panel labeled as 'EBM84-H' mistakenly displayed the image corresponding to the 'EBM84-M' group. The revised version of Fig. 4, now showing the correct data for the 'Mon', 'EBM84-M' and 'EBM84-H' data panels in Fig. 4B, is shown on the next page. The authors confirm that the errors made during the assembly of this figure did not have a significant impact on either the results or the conclusions reported in this study, and all the authors agree with the publication of this Corrigendum. The authors are grateful to the Editor of *International Journal of Molecular Medicine* for allowing them the opportunity to publish this Corrigendum; furthermore, they apologize to the readership of the Journal for any inconvenience caused.

## Figures and Tables

**Figure 4:**
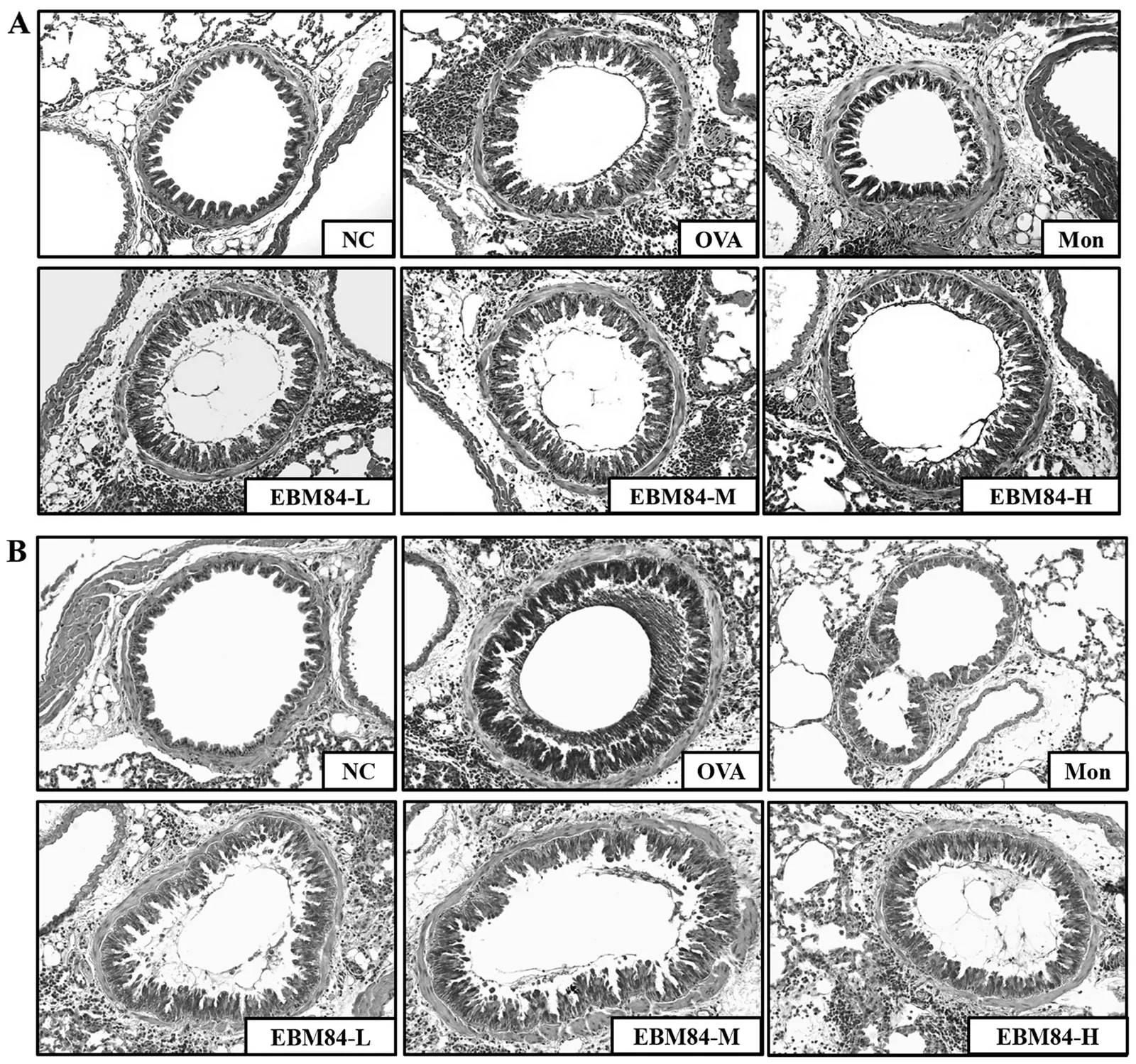
EBM84 inhibits the recruitment of inflammatory cells to the lung tissues of mice 48 h after the final OVA challenge. (A) Histological examination of lung tissues with hematoxylin and eosin staining (magnification, ×200). (B) Histological examination of mucus secretion in lung tissue with periodic acid Schiff staining (magnification, ×200). EBM84 reduced airway inflammation in peribronchiolar and perivascular lesions, as well as mucus hypersecretion induced by OVA challenge.

